# Indicators of Impulsivity in Routine Clinical Assessment of Adult ADHD

**DOI:** 10.1177/10731911251365744

**Published:** 2025-09-16

**Authors:** Hui Dong, Anselm B.M. Fuermaier, Janneke Koerts, Gerdina H.M. Pijnenborg, Nana Guo, Ragnar Schwierczok, Norbert Scherbaum, Bernhard W. Müller

**Affiliations:** 1 University of Groningen, The Netherlands; 2 Henan Normal University, Xinxiang, China; 3 University of Duisburg-Essen, Germany; 4 University of Wuppertal, Germany

**Keywords:** impulsivity, commission errors, reaction time, self-reports, factor analyses, factor structure

## Abstract

Impulsivity in adult attention-deficit/hyperactivity disorder (ADHD) represents a multidimensional construct rather than a unitary trait. This study examined a proposed three-factor model of impulsivity comprising (a) self-reported impulsive behavior (Barratt Impulsiveness Scale), (b) commission errors, and (c) reaction time measures from neuropsychological tests in 654 adults undergoing routine clinical assessment of adult ADHD. Using confirmatory factor analyses on split subsamples, we found consistent support for the proposed three-factor structure, whereas the network analysis favored a two-group conceptualization that separates performance-based from self-report-based measures. Self-reported impulsivity demonstrated the highest severity levels, followed by commission errors, with reaction times being least affected. Demographic and clinical characteristics significantly predicted self-reports and commission error measures but not reaction times. The results emphasize the importance of interpreting self-reports independently of performance-based tests. The coherence between commission errors and reaction time variables across tasks of related constructs suggests that administering multiple tasks may yield redundant information in the clinical assessment of impulsivity.

## Introduction

Impulsivity is a primary DSM-5 (*Diagnostic and Statistical Manual of Mental Disorders;* American Psychiatric Association, 2013) diagnostic feature of attention-deficit/hyperactivity disorder (ADHD) that is associated with several undesirable consequences, such as social/peer relational problems (Diamantopoulou et al., 2007), academic failure (Merrell & Tymms, 2001), financial mismanagement (Koerts et al., 2023), and criminal conviction (Moffitt et al., 2011). A high level of impulsivity that persists into adulthood is associated with a range of adult psychiatric disorders, including mania (Strakowski et al., 2009, 2010), substance abuse disorder (Verdejo-García et al., 2008), personality disorders (Linhartová et al., 2020), eating disorders (Waxman, 2009), and pathological gambling (Grall-Bronnec et al., 2012). As such, impulsivity may function as a transdiagnostic risk factor for a broad range of psychopathologies.

Impulsivity is a multifaceted construct with numerous definitions and related concepts (Evenden, 1999; Mitchell & Potenza, 2014). It can be broadly defined as both a long-term behavioral trait (i.e., trait-dependent component) and a transient state (i.e., state-dependent component) characterized by a tendency to act prematurely without foresight (Barnhart & Buelow, 2017). The trait-dependent component is usually assessed through (personality) self-report inventories, while the state-dependent component is commonly assessed through neuropsychological performance tests (behavioral measures). Studies on the relationship between measures of trait-dependent and state-dependent components of impulsivity have yielded mixed results, with some studies reporting significant associations (Hamilton et al., 2014; Leshem & Glicksohn, 2012; Meule, 2013), while the majority of research has failed to find significant and meaningful relationships (Barnhart & Buelow, 2017; Fino et al., 2014; Meda et al., 2009). This observed discrepancy between self-reports and performance tests is a common phenomenon in cognitive assessments in which subjective self-ratings and objective performance tests do not always align (e.g., as discussed by Fuermaier et al., 2015). Moreover, studies using factor- and meta-analysis techniques advocated a multifaceted nature of impulsivity, with the exact number of factors depending on the number and types of measures (Barnhart & Buelow, 2017; Ortal et al., 2015; Sharma et al., 2014; Whiteside & Lynam, 2001). Research spanning a broad range of measures on non-clinical and clinical populations, including ADHD across the lifespan, so far, has not reached a consensus on the best measurement of impulsivity but supports the notion that multiple approaches and indicators of impulsive behavior should be considered. However, the broad choice of instruments and unclear conceptualization can be challenging for clinicians and researchers to select and interpret impulsivity measures in their given context.

Regarding self-report measures, a number of multifaceted tools have been introduced that were shown to effectively capture various dimensions of impulsivity in adults with ADHD, such as the UPPS Impulsive Behavior Scale (UPPS; Whiteside & Lynam, 2001), the Barratt Impulsiveness Scale (BIS-11; Felthous & Barratt, 2003), or ADHD symptom scales for a dimensional assessment of the severity of impulsive behavior (Conners, Erhardt, Epstein, et al., 1999; Dickman, 1990; Kessler et al., 2005). Specific measures give a more nuanced differentiation between dimensions of impulsivity, as defined in the UPPS (i.e., urgency, lack of premeditation, lack of perseverance, and sensation seeking) or in the BIS-11 (i.e., action without thinking, lack of focus on the task at hand, and non-planning). A large extent of research demonstrated higher levels of impulsivity in all mentioned aspects in individuals with ADHD compared to their typically developing peers (e.g., Linhartová et al., 2020; Lopez et al., 2015; Malloy-Diniz et al., 2007; Nandagopal et al., 2011). However, differential effects were observed when ADHD was compared to other psychiatric conditions (e.g., higher levels of non-planning impulsivity in individuals with bipolar disorder than in individuals with ADHD, see Nandagopal et al., 2011).

In contrast to trait levels of impulsivity as assessed by self-report instruments, neuropsychological performance tests conceptualize impulsivity as in-the-moment, state-like, rash actions in a variety of behavioral paradigms. The inability to withhold from making a response, also known as the rapid-response impulsivity that sacrifices accuracy for speed (Evenden, 1999), is commonly assessed with neuropsychological measures in ADHD and related conditions, with such variants of a Go/No-task (Drewe, 1975), Stop-Signal task, Stroop task (Stroop, 1935), Flanker task (Jonkman et al., 1999), or Card playing/Door opening task (Daugherty & Quay, 1991). What these paradigms have in common is that they measure the inability to inhibit a prepotent response that is thought to be one of the fundamental deficits that characterize ADHD. A wealth of studies has revealed a significant slowing of reaction time and an increased number of impulsive errors in individuals with ADHD in the above-mentioned impulsivity tasks compared to non-affected controls (Lijffijt et al., 2005; Metin et al., 2012; Roberts et al., 2016). Despite the clear strengths of neuropsychological tests as objective, in-the-moment performance indicators, the results are often challenging to interpret, as neuropsychological functions are intertwined and are directly and indirectly related to a range of cognitive and non-cognitive parameters, including test motivation, attention and concentration, working memory, planning, and flexibility (Dougherty et al., 2002; Guo et al., 2022). Furthermore, the choice and interpretation of suitable measures for rapid-response impulsivity are complicated, as similar information can also be derived from other clinical tests, such as variants from the widespread Continuous Performance Tests (CPTs; Avila et al., 2004; Conners, 2014; Swann et al., 2002). CPTs are computerized tests for different aspects of attention (primarily selective attention and vigilance), which require individuals to respond to predefined target stimuli that are presented over a relatively long and unbroken period of time. In addition to measures of reaction time and omission errors (i.e., the number of times the participant failed to respond when a target was presented), which indicate distractibility, commission errors (CE; i.e., responding to non-targets) can be interpreted as measures of impulsivity. Evidence from numerous studies has shown that individuals diagnosed with ADHD make more errors of commission on CPTs (Huang-Pollock et al., 2012; Kuntsi et al., 2001; Winstanley et al., 2004), which can be interpreted as increased levels of impulsivity. Thus, CPTs are commonly used in pediatric neuropsychology as a standardized tool to assess various aspects of attention and impulsivity (Barnard et al., 2018; Berger et al., 2017; López-Vicente et al., 2014) and have also gained popularity in adult assessment settings (Fuermaier et al., 2019, 2023; Huang-Pollock et al., 2012).

The literature reviewed above indicates that state-like impulsivity can be assessed through various specific neuropsychological tests (such as interference and Go/No-Go tasks), but can also be derived from tests for other cognitive functions (e.g., selective attention and vigilance). Across most of the tasks, commission errors and reaction times seem to stand out consistently as primary performance indicators of impulsivity. Although both measures are commonly used as indicators of state-like impulsivity, they are theorized to reflect distinct components of attentional processing. Reaction times index the speed of information processing and response execution, while commission errors reflect premature or incorrect activation of responses, associated with failures in selective attention or inhibitory control (Kofler et al., 2013). Faster reaction times have been linked to increased commission error rates, suggesting a trade-off between speed and control in impulsive individuals (see Heitz, 2014, for a review). Together with self- and observer-report measures, a breadth of impulsivity assessments is available to clinicians and researchers, who may experience challenges in selecting and interpreting impulsivity measures appropriately.

In this study, we aim to bring clarity to the interpretation of impulsivity measures in routine clinical neuropsychological evaluations of adult ADHD and related disorders. Based on an extensive body of research on self-reports and neuropsychological performance tests (Cyders & Coskunpinar, 2012; Kräplin et al., 2014; Reynolds et al., 2008), we assume that self-reported information on impulsivity is distinct from neuropsychological performance data on impulsivity. Furthermore, in batteries of rapid-response tasks that require a trade-off between speed and accuracy, we have reasons to assume that errors of commission across different tests represent a distinct factor of impulsivity, which is different from slowed-down responses in impulsivity measures (Dougherty et al., 2002; Halperin et al., 1991; Weafer et al., 2013). While prior review studies (e.g., Cyders & Coskunpinar, 2011; Sharma et al., 2014) have established distinctions between behavioral and self-report measures, our study extends this by empirically testing whether commission errors consistently load on a single latent factor across tasks, and whether reaction time metrics form a coherent factor. Crucially, we combine factor analysis with network modeling to explore both the latent structure and the interconnectedness of impulsivity indicators, offering a more nuanced understanding of their relationships. Thus, we examine the presence of a three-factor structure of impulsivity within this naturalistic setting, which encompasses (a) self-report impulsivity measures from questionnaires, (b) commission errors, and (c) reaction time metrics across neuropsychological performance tests spanning different aspects of impulsivity. To achieve this, we employ a combination of network and factor analysis techniques on independent samples to examine both the interrelationships (capturing distinctions and potential redundancies) and the factorial structure of various impulsivity measures drawn from a large cohort of individuals clinically referred to an ADHD outpatient clinic.

The primary goal of this study is to establish a robust factor structure and, by doing so, add clarity to the assessment of impulsivity in routine neuropsychological assessments of adults with ADHD. In addition, we will examine potentially influential factors affecting the robustness of this factor structure by analyzing the impact of suspicious neuropsychological data (Dong et al., 2023; Guo et al., 2022). Furthermore, we will consider a wide range of demographic and clinical characteristics, including age, sex, ADHD diagnostic status, and symptom severity, as well as comorbidities such as addiction or mood disorders and related constructs like sensation seeking to explore their associations with different factors of impulsivity. The implications of our findings for assessing impulsivity in individuals with ADHD and related disorders will also be discussed.

## Methods

### Participants

All participants were suspected to suffer from ADHD in adulthood and were, therefore, referred for a comprehensive diagnostic evaluation to the ADHD outpatient clinic at the [Department of Psychiatry and Psychotherapy, University of Duisburg-Essen, Germany]. The diagnostic assessment procedure adhered to the criteria outlined in *DSM-5* (American Psychiatric Association, 2013) and followed empirically informed diagnostic procedures for first-time adulthood ADHD (Sibley, 2021). The core feature of the diagnostic procedure was semi-structured interviews to evaluate ADHD psychopathology, that is, the Wender-Reimherr Interview (Retz-Junginger et al., 2017) and the Essen-Interview-for-schooldays-related-biography (Grabemann et al., 2017). The diagnostic interviews were complemented with a battery of self- and informant-report rating scales. The Conners’ Adult ADHD Rating Scales (CAARS) were applied as a self- and informant report, to assess current ADHD symptom severity (Conners, Erhardt, & Sparrow, 1999). Furthermore, the CAARS has been shown to identify possible symptom overreporting using suggested cut scores on normative T-scores (Conners, Erhardt, & Sparrow, 1999). In addition to symptom criteria, the diagnostic assessment included an evaluation of impairments in major life domains (e.g., academic and/or occupational functioning), and evidence for alternative explanations to ADHD. In the diagnostic procedure, clinicians gathered information from multiple sources, including medical files, reports from schools, employers, and reports provided by partners or parents. Finally, a comprehensive neuropsychological assessment with cognitive tests completed the diagnostic evaluation. Comorbid psychiatric disorders were identified based on prior clinical documentation (e.g., psychiatric records or discharge summaries), current clinical interviews, and questionnaires. While no structured diagnostic interview for the full range of psychiatric conditions was used, additional assessments (i.e., autism questionnaires) were conducted when clinically indicated. All patients were seen by both a licensed psychiatrist and a clinical psychologist, and comorbidity-related questions in complex cases were discussed in interdisciplinary case conferences. Overall, the final diagnoses—including the diagnosis of ADHD—were made by trained professionals based on clinical judgment, integrating all available information rather than relying on fixed cutoff scores.

For the present study, we retrieved a data set of 745 participants, whom we considered for inclusion in the present study. Missing data occurred in 12% of the cases and resulted in the removal from all analyses, leaving a sample of 654 participants who were included in the final data analyses. To evaluate whether this exclusion introduced systematic bias, we compared participants with complete data (*N* = 654) to those with missing data (*N* = 91) on key demographic and clinical variables, including age, sex, and ADHD symptom severity (three DSM scales). Pearson’s chi-square tests and Wilcoxon rank-sum tests revealed no significant differences between the two groups (all *p* > .05), suggesting that the missingness was likely random and not associated with core study variables. Three hundred and fifty-one adults (53.7%) were diagnosed with ADHD after a comprehensive evaluation (ADHD group). One hundred and twenty-one adults with ADHD were diagnosed with at least one comorbid psychiatric disorder, with mood disorders and/or addiction disorders being most often established (see Table 1 for details). A total of 303 participants (46.3%, n-ADHD group) did not fulfill the diagnostic criteria for ADHD, and about half of them were diagnosed with a psychiatric disorder other than ADHD. Mood disorders and addiction disorders were, again, most often diagnosed. Demographic variables and group characteristics are presented in Table 1. Significant differences were observed between the ADHD and n-ADHD group in terms of sex (χ^2^ = 9.7, *p* = .002) and age (*Z* = −3.7, *p* < .001), but not in educational level (χ^2^ = 6.8, *p* = .149). As expected, individuals with ADHD reported significantly higher scores in current self-reported ADHD symptoms as measured by the three DSM subscales of the CAARS, that is, inattention (*Z* = −3.4, *p* = .001), hyperactivity–impulsivity (*Z* = −4.4, *p* < .001), and total ADHD symptoms (*Z* = −4.6, *p* < .001).

**Table 1.** Group Characteristics and Clinical Information (Total Sample, *N* = 654).

Variables	ADHD (*n* = 351)	*n*-ADHD (*n* = 303)	*Z/*χ^2^	*p*
Sex			9.7	.002*
- Male	222 (63.2%)	155 (51.2%)		
- Female	129 (36.8%)	148 (48.8%)		
Age (in years) ^a^	32.0 ± 8.9	35.5 ± 11.2	−3.7	< .001**
Education (5 levels) ^b^	15/56/104/111/64/1	3/46/94/100/59/1	6.8	.149
Comorbidity (Yes/No)	110/241 (31.3%/68.7%)	48/255 (15.8%/84.2%)		
Psychiatric disorders other than ADHD ^d^	68/24/10/13/0/5/2/3/1/2/5/2/0/2/1	107/26/13/16/5/13/3/6/1/1/4/1/1/1		
Symptom presentation of ADHD ^c^	300/3/48 (85.5%/0.9%/13.7%)			
Current ADHD symptom severity ^e^				
Inattention	18.59 ± 7.49	17.01 ± 5.41	−3.4	.001
Hyperactivity-impulsivity	13.64 ± 5.86	11.62 ± 6.02	−4.4	<.001**
Total ADHD symptoms	31.79 ± 9.25	28.50 ± 9.84	−4.6	<.001**

*Note.* ADHD = attention-deficit/hyperactivity disorder.

a Data were missing for two participants in the ADHD group.

b Education = Basic schooling with no formal degree (less than 9 years of schooling)/Secondary school (usually 9–10 years of schooling)/Secondary school with additional vocational training (usually 10–12 years of schooling)/Secondary school with university entrance qualification (usually 12–13 years of schooling)/University degree (usually 16–17 years of schooling)/Not reported.

c Symptom presentation of ADHD = combined/inattentive/hyperactive-impulsive/not reported.

d Psychiatric disorders other than ADHD = mood disorders/addiction disorders/anxiety disorders/personality disorders/eating disorders/adjustment disorders/schizoaffective disorders/obsessive-compulsive disorders/conduct disorders/intellectual developmental disorders/developmental disorders/habit and impulse disorders/post-traumatic stress disorders/other disorders.

e Measured with the Conners’ Adult ADHD Rating Scales—Self-Report (three DSM scales): Long Version (CAARS-S: L).

Statistically significant at *p* < .05*, *p* < .001**.

### Measures

For the present study, we selected measures from our routine assessment battery that assess one or several aspects of impulsivity. Our selection was based on seminal previous conceptual and empirical work on impulsivity measures in this clinical assessment context (Dougherty et al., 1999; Kjome et al., 2010; Preuss et al., 2008; Sanchez-Roige et al., 2012). Tests and questionnaires for the assessment of impulsivity are presented below. The measures include commission errors of tests for selective attention, sustained attention, and a Go/No-Go task, timed variables of measures for interference control (i.e., Stroop) and inhibitory control (i.e., Go/No-Go), and self-report instruments on impulsive behavior. The variables per instrument, together with the allocation to the various factor models, are presented in Table 2.

**Table 2.** Measures of Impulsivity and Item Allocation to Factor Structures (Total Sample, *N* = 654).

Measures	Range (min, max)	Mean	Median	*SD*	% below average ^a^	Conceptual three-factor model	Post hoc three-factor model	Conceptual two-factor model	Conceptual one-factor model
WAFS_CE	0, 96	4.71	3	7.39	14.1	Commission errors	Commission errors	Test performance	One factor
WAFV_CE	0, 154	4.72	3	10.57	31.8	Commission errors	Commission errors	Test performance	One factor
INHIB_CE	0, 34	14.89	15	7.02	22.2	Commission errors	Commission errors	Test performance	One factor
INHIB_RT	0.15, 3.24	0.29	0.28	0.13	7.2	Reaction time	Commission errors	Test performance	One factor
STROOP_RI	−0.06, 1.65	0.21	0.17	0.17	17.0	Reaction time	Reaction time	Test performance	One factor
STROOP_NI	−0.07, 1.05	0.14	0.11	0.12	6.4	Reaction time	Reaction time	Test performance	One factor
BIS_AI	4, 37	22.11	22	4.06	94.2	Symptom reports	Symptom reports	Symptom reports	One factor
BIS_MI	12, 40	26.14	26	5.35	88.2	Symptom reports	Symptom reports	Symptom reports	One factor
BIS_NI	12, 94	29.73	30	6.16	25.7	Symptom reports	Symptom reports	Symptom reports	One factor

a *Note.* Below average is defined as PR ≤ 8 on neuropsychological tests and PR ≥ 92 on questionnaires scores, each based on healthy normative groups. WAFS_CE = Perceptual and Attention Functions Test (Selective)_ Commission errors; WAFV_CE = Perceptual and Attention Functions Test (Vigilance)_Commission errors; INHIB_CE= Go/No-Go test _Commission errors; INHIB_RT = Go/No-Go test _ Reaction time; STROOP_RI = Stroop interference test _Reading-interference; STROOP_NI = Stroop interference test _Naming-interference; BIS = Barratt Impulsivity Scale, BIS_AI = attentional impulsivity, BIS_MI = motor impulsivity, BIS_NI = non-planning impulsivity.

**Table 3.** One-, Two-, and Three-Factor CFA Model Fit Statistics on Subsample 2 (*n* = 375).

Model	χ2	χ2/*df*	RMSEA	CFI	TLI
Three-factor model	38.001	1.583	0.046	0.932	0.898
Three-factor model (post hoc)	41.370	1.724	0.049	0.924	0.887
Two-factor model	56.260	2.164	0.057	0.889	0.846
One-factor model	112.802	4.178	0.088	0.725	0.634

*Note*. CFA = confirmatory factor analysis; RMSEA = root-mean-square error of approximation; CFI = comparative fit index; TLI = Tucker–Lewis index; Model-data fit is considered acceptable when χ2/*df* ranges from 2 to 5, RMSEA is below 0.06, and CFI and TLI estimates exceed 0.80; A fit is considered good when χ2/*df* close to 1, and CFI, TLI estimates surpass 0.95 (Hox et al., 2017).

#### BIS-11, German version

The German version of the Barratt Impulsivity Scale is a 30-item self-rating tool derived from the original items of the English BIS-11 (Preuss et al., 2008). Each item addresses impulsivity-related thoughts or behaviors in various situations, and participants rate the frequency of these experiences on a 4-point scale: 1 = “*Rarely/never*,” 2 = “*Occasionally*,” 3 = “*Often*,” and 4 = “*Almost always/always*.” The BIS has been extensively used in clinical research and practice over the last decades (e.g., see Maraz et al., 2016; Stanford et al., 2009), including studies of clinical samples with ADHD (Roberts et al., 2016). The BIS-11 conceptualizes impulsivity as a multidimensional construct, encompassing motor, attentional, and non-planning impulsivity. In each of these dimensions, higher scores indicate greater levels of impulsivity. Published reliability coefficients (Cronbach’s alpha) for the BIS-11 range from .74 to 0.83 (Preuss et al., 2008). The presence of impulsivity was evaluated based on a normative sample of the healthy population (*n* = 810, mean age = 47, 53.7% women), with mean scores for motor impulsivity at 15.62 (*SD* = 2.49), attentional impulsivity at 13.68 (*SD* = 1.40), and non-planning impulsivity at 28.82 (*SD* = 3.37).

#### Selective Attention (WAFS, Commission Errors)

The Perceptual and Attention Functions—selective attention (WAFS; (Sturm, 2011) is a test for selective attention and impulsivity, administered on the Vienna Test System (Schuhfried, 2013). In recent research, the WAFS was also studied as an embedded validity indicator (Dong et al., 2023; Guo, Fuermaier, Koerts, Mueller, Diers, et al., 2021). The stimuli of this test were presented sequentially on a computer screen. Participants were instructed to respond only to relevant stimuli by pressing the response key as quickly as possible and to ignore irrelevant stimuli. Impulsive behavior was assessed by tracking the number of commission errors, defined as reactions occurring without a preceding stimulus or in response to an irrelevant stimulus. The performance on the WAFS was evaluated using a normative group of 295 individuals from the general population, aged 16 to 77 (median = 39, *SD* = 15.1), with a gender distribution of 46.4% men and 53.6% women. All participants in the normative group completed the tests in German. The internal consistency of this test is excellent, with a Cronbach’s α of .95 in the normative sample.

#### Vigilance (WAFV, Commission Errors)

The Perceptual and Attention Functions—vigilance (WAFV; Sturm, 2012) is a test for sustained attention (vigilance) and impulsivity, administered on the Vienna Test System (Schuhfried, 2013). In recent research, this test has also been shown to be effective in distinguishing credible from non-credible cognitive performance in individuals with ADHD (Becke et al., 2023). Participants were instructed to respond as quickly as possible by pressing the response key upon detecting the target stimulus. Impulsivity was assessed by recording the number of commission errors, which indicate responses to false or non-existent stimuli. The internal consistency (Cronbach’s α) of the main variables was reported to be .96. Individual scores were evaluated on the same German-speaking normative sample described for the WAFS (*N* = 295, aged 16 to 77 years, 53.6% women).

#### Go/No-Go (INHIB; Reaction Times and Commission Errors)

The Go/No-Go is a test for inhibitory control (Drewe, 1975), administered on the Vienna Test System labeled as INHIB (Kaiser et al., 2016). Participants are instructed to press a response button whenever the target stimulus appears but to refrain from responding when a non-target stimulus is shown. Impulsivity was measured by recording both the number of commission errors and the mean reaction time (RT in ms). The normative sample comprised 359 adults, including 175 males (48.7%) and 184 females (51.3%), with a mean age of 44.07 years (*SD* = 17.60), ranging from 16 to 84 years. The parameter of commission errors reports the number of false reactions in stop trials. This test’s internal consistency (Cronbach’s α) was reported to be .83 in the normative sample.

#### Stroop (Reaction Times)

The Stroop interference test was applied as a measure of interference control (Schuhfried, 2016), which goes back to the original version of Stroop (Stroop, 1935). The key conditions of this task involved reading interference and naming interference. In the reading-interference condition, participants had to respond to the meaning of color words (e.g., BLUE, GREEN, YELLOW, RED) while ignoring the color in which the word was printed (e.g., the word GREEN printed in red ink). Conversely, in the naming-interference condition, participants needed to react to the color of the word while disregarding its meaning. Impulsivity was evaluated using raw reaction times in seconds for reading and naming-interference tasks. Standardization was conducted using a representative sample of *N* = 327, comprising 47.7% males and 52.3% females, with a mean age of 41.9 years and a standard deviation of 18 years. The internal consistency (Cronbach’s α) of the main variables (i.e., the primary outcome measures defined in standardized test manuals to capture the core cognitive functions targeted by each neuropsychological task) was reported to be .97.

#### Validity Assessment

Based on recent research on validity assessment in adults with ADHD, we determined possible non-credible performance and symptom reporting using the WAFV and CAARS. For performance validity, we applied the cutoff of WAFV-omissions ≥ 7, as suggested by Becke et al. (2023), who demonstrated its suitability as a validity measure particularly under low base rate conditions. For symptom validity, we used CAARS-based criteria: DSM total T scores ≥ 80 (indicative of probable overreporting) and inconsistency scores ≥ 8 (indicative of possible symptom inconsistency). We implemented a tiered decision strategy by separately excluding participants who met criteria for probable underperformance, probable inconsistent symptom reporting, and probable symptom overreporting. We did not exclude participants falling above these cutoffs in our main analysis, as we had no solid and generally accepted criterion for invalid data (such as the two-failure rule for cognitive performance tests, see Sweet et al., 2021). Instead, we repeated our main analysis on the three data subsets to explore the influence of probable invalid data in additional and exploratory analyses.

### Procedure

The protocol of this study received ethical approval from the medical ethical review board of the [blinded for review]. Written informed consent was obtained from all participants who agreed to the use of their routine clinical data for research purposes. Self- and other-report questionnaires were completed at home prior to the evaluation. On the day of the assessment, clinical interviews were conducted, together with a review of medical records and a comprehensive neuropsychological assessment with cognitive tests on the same day or another. The neuropsychological assessment was administered by trained psychologists or psychological test assistants under supervision and took about 2 hr to complete (see “Methods” for details). The data set used for this research project stems from a consecutively collected large-scale data set of the [blinded for review], which aims to address diverse topics in the field of ADHD and related disorders. Parts of the data have therefore already been used for previous projects on different research questions (Dong et al., 2023; Guo, Fuermaier, Koerts, Mueller, Diers, et al., 2021; Guo, Fuermaier, Koerts, Mueller, Mette, et al., 2021; Guo et al., 2022).

### Statistical Analysis

The goal of the analysis was to present, compare, and evaluate the factor structures of impulsivity measures from this routine clinical neuropsychological assessment. Conceptually derived and supported by previous research, we assume a three-factor structure with self-reports, commission errors, and timed test variables, forming three factors. Competing models are a one-factor model and a two-factor model (with performance tests and self-reports forming the factors). We planned to conduct a two-step analysis: first, a network analysis to explore the coherence of the measures in a visual presentation, and, on an independent sample, a confirmatory factor analysis (CFA) to validate the findings. Moreover, network analysis brought up an alternative three-factor model, which we considered in factor analysis (see Table 2 for an overview and item allocation). All factor analyses were performed using R version 4.3.1.

#### Sample Size

To enhance the generalizability of our findings, we strived for network analysis and CFA on different samples. In factor analyses, sample size determination is considered an important issue to achieve sufficient power for detecting hypothesized indirect effects (Costello & Osborne, 2019). For this reason, we conducted a priori power analysis to estimate the required sample size for CFA based on the following parameters: degrees of freedom = 24, RMSEA ≥ 0.05, alpha level = .05, and desired statistical power = 80%, (semPower package in R, see Moshagen and Bader, 2024 for the code). Power computation indicated a minimum required sample size of 375 participants for CFA. To meet these analytical requirements, the entire dataset (*N* = 654) was randomly divided into two subsamples, that is, subsample 1 (279 participants) for network analysis and subsample 2 (375 participants) for CFA. Sample size determination for network analysis is less straightforward; however, the larger the samples, the more stable and more accurate the network estimates that are obtained (Hevey, 2018). The consequences of the sample size selection for the stability of the network analysis are discussed in the “Results” and “Discussion” sections.

#### Descriptive Statistics and Univariate Comparisons

Group characteristics and clinical information of the total sample are presented descriptively and compared between the ADHD and n-ADHD groups using non-parametric statistics (i.e., Mann–Whitney U tests). Furthermore, impulsivity measures are presented in descriptive statistics and compared to test norms, that is, showing the proportion of individuals with “below average” performance and self-report. “Below average” is defined by Guilmette et al. (2020) with a percentile rank (PR) ≤ 8 on tests and PR ≥ 92 on self-report scales.

#### Network Analysis

To visually and structurally elucidate the interrelationships among various variables involved in impulsivity measures, a network analysis was employed. Network analysis comprises three core analytical steps: the initial step is the estimation of the network, followed by the estimation of node centrality, and culminating with the assessment of accuracy and stability. First, network estimation visually represents the pattern of relationships between variables and a network can be estimated using partial correlation that quantifies relationships. The R package “*bootnet”* was used to construct networks (Epskamp et al., 2018; Epskamp et al., 2012) using Graphical Gaussian Models with the Least Absolute Shrinkage Operator (GLASSO; Friedman et al., 2008) and extended Bayesian Information Criterion model selection (Foygel & Drton, 2010). To eliminate spurious connections and enhance interpretability, the “Least Absolute Shrinkage and Selection Operator” (LASSO; Tibshirani, 1996) regularization technique was used. Network visualization used the Fruchterman-Reingold algorithm. To enhance interpretability, nodes in the network visualization were color-coded according to their hypothesized factor structure: commission errors factor (nodes 1–3) in red, reaction times factor (nodes 4–6) in green, and self-reports factor (nodes 7–9) in purple. This conceptual color coding was used to visually assess whether variables associated with the same latent factor cluster together in the network structure. For a detailed description of the analytic procedures, see tutorials and reviews by Costantini and Epskamp (2017) and Epskamp et al. (2018). Given that our data were not normally distributed, a rank transformation (Spearman correlations as input) was performed before estimating the network structures (Isvoranu & Epskamp, 2021). Second, we assessed the centrality of each item, which reflects the extent to which a given item is interconnected with all other items within the network using node expected influence, a centrality index representing the sum of connections for each node. The *centrality, centralityTable*, and *centralityPlot* functions of the *“qgraph”* package were utilized to compute and visualize expected influence (Epskamp et al., 2012). These analyses identified whether items in the network clustered together in a particular manner. Lastly, bootstrapping was employed to estimate the 95% confidence intervals (CIs) of the edge weights to evaluate the accuracy of edge weights and the stability of node centrality rankings. Smaller CIs indicate higher accuracy for most edges in the network. Node centrality stability was evaluated using the correlation stability coefficients (CS coefficients), with coefficients greater than .25 indicating moderate stability and those greater than .5 indicating strong stability, utilizing the R package “*bootnet”* for these analyses.

#### Confirmatory Factor Analysis

In addition to network analysis, CFA was conducted on subsample 2 of *n* = 375 to test the expected factor structure of impulsivity in adults with ADHD. CFA results confirmed the factor structure visually identified in the network analysis, providing evidence of construct validity in theory-based instrument construction and development (Kline, 2023). Given that our selected nine variables encompass both count and continuous data (reaction times and test scores), and considering the presence of missing data in our dataset, we employed the Robust Maximum Likelihood estimator. This estimator was chosen because it can correct for non-normality in the dataset and is suited for handling mixed-type data (Kilic & Doğan, 2021; Lubke & Muthén, 2004). In addition, we standardized all items to ensure comparability and improve model stability. Specifically, we applied z-score standardization (mean = 0, *SD* = 1) to the nine continuous variables. Global fit statistics were used to assess the validity of the models, including the standard chi-square statistic (χ^2^/*df*), robust root mean square error of approximation (RMSEA), robust confirmatory fit index (CFI), and robust Tucker–Lewis index (TLI) (Brown, 2015). Consistent with structural units summarized in previous related studies (Barnhart & Buelow, 2017; Sharma et al., 2014), this study specified three models with one (all items), two (self-reports, test performance), and three (self-reports, commission errors, and reaction times) latent factors, respectively (see Table 2 for item allocation to the different factor models). Because these models were statistically nested, we conducted chi-square comparisons among the three models (e.g., one-factor vs. two-factor vs. three-factor model comparisons) to assess their statistical differences.

In addition to post hoc analyses, based on the results of network analysis, we included another three-factor structure in our model comparisons. Specifically, the reaction time of the Go/No-Go tests was reallocated to the factor that includes commission errors, as indicated in the network structure. We further compared the fit of both three-factor models. For this, the difference between the Bayesian Information Criterion (BIC) for two models (△BIC) can be computed, and the lower BIC indicates a better fit (McCoach & Black, 2008). Following Raftery (1995), the △BIC is interpreted as follows: 0 ≤ △BIC < 2 (weak evidence for difference), 2 ≤△BIC < 6 (positive evidence for difference), 6 ≤ △BIC < 10 (strong evidence for difference), and △BIC ≥ 10 (very strong evidence for difference) (Anderson, 2002).

#### Influence of Potential Invalid Data on Network Analysis and Confirmatory Factor Analysis

Network analysis and CFA were repeated on the three subsets after removing all cases with probable underperformed test results, probable inconsistently reported symptoms, and probable overreported symptoms.

#### Multiple Linear Regression

The *lavPredict* function of the *“lavaan”* package generated scores for each observation on each latent factor. These factor scores represented impulsivity indicators which are used as criteria in regression models. Three linear regression models were computed based on a range of descriptive and clinical variables (Table 4) predicting impulsive behavior as indicated by the three-factor scores. Variance inflation factors (VIFs) were computed for all three regression models to assess multicollinearity among predictors. All VIFs ranged between 1.03 and 1.34, well below the commonly accepted threshold of 2.5, indicating that multicollinearity was not a concern in any of the models (Midi et al., 2010). This suggests that the estimated regression coefficients were not distorted by redundant predictor information, and the predictors contributed independently to the explanation of variance in impulsive behavior.

**Table 4.** Multiple Linear Regression Models Based on Descriptive and Clinical Information to Predict Impulsivity as Indicated in the Three Factor Scores (Total Sample, *N* = 654).

Predictors	β	*SE*	*t*	*p*	*F-*value	*p*	*R* ^2^	Adjusted *R*^2^
**Model 1 (Commission errors)**								
					4.15	*<* .001**	.060	.046
Age	−.0084	.0086	−.973	.331			.	
Sex (male/female)	.2024	.1720	1.177	.240				
ADHD (no/yes)	.2457	.1715	1.433	.153				
Addiction disorders (no/yes)	.0079	.3714	.021	.983				
Mood disorders (no/yes)	.3032	.2010	1.509	.132				
Inattention symptoms	.0159	.0130	1.227	.221				
Hyperactive-impulsive symptoms	.0595	.0158	3.762	< .001**				
Sensation seeking	−.0637	.0754	−.844	.399				
**Model 2 (Reaction time)**								
					1.05	.401	.016	.001
Age	.0002	.0005	.585	.559				
Sex (male/female)	−.0109	.0098	−1.105	.270				
ADHD (no/yes)	−.0001	.0098	−.008	.994				
Addiction disorders (no/yes)	−.0087	.0212	−.409	.683				
Mood disorders (no/yes)	.0153	.0115	1.322	.184				
Inattention symptoms	.0015	.0007	1.983	.048*				
Hyperactive-impulsive symptoms	−.0005	.0009	−.599	.550				
Sensation seeking	−.0032	.0043	−.735	.463				
**Model 3 (Self-reports)**								
					55.38	*<* .001**	.462	.453
Age	−.0048	.0019	−2.503	.013				
Sex (male/female)	.1009	.0380	2.652	.008*				
ADHD (no/yes)	.0597	.0379	1.574	.116				
Addiction disorders (no/yes)	.0878	.0822	1.069	.286				
Mood disorders (no/yes)	.0126	.0445	0.284	.777				
Inattention symptoms^a^	.0212	.0029	7.392	< .001**				
Hyperactive-impulsive symptoms^a^	.0427	.0035	12.195	< .001**				
Sensation seeking^b^	.0671	.0167	4.022	< .001**				

a Inattention symptoms and hyperactive-impulsive symptoms were measured with the Conners’ Adult ADHD Rating Scales—Self-Report (three DSM scales): Long Version (CAARS-S: L).

b Sensation seeking was assessed using the Sensation-Seeking Need Inventory.

Statistically significant at p < .05*, p < .001**.

## Results

### Descriptive Statistics and Univariate Comparisons

**Figure 1.**
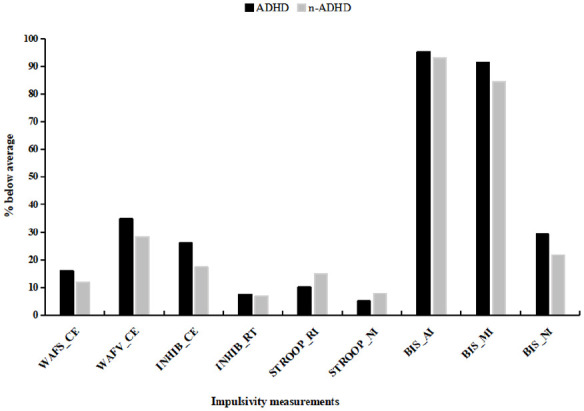
Proportion of Impulsivity Measurements in Neuropsychological Test Performance and Self-Report for the Entire Sample (*n* = 654). *Note.* Below average is defined as PR ≤ 8 on neuropsychological tests and PR ≥ 92 on questionnaires scores, each based on healthy normative groups. WAFS_CE = Perceptual and Attention Functions Test (Selective Attention)_ Commission errors; WAFV_CE = Perceptual and Attention Functions Test (Vigilance)_Commission errors; INHIB_CE= Go/No-Go test _Commission errors; INHIB_RT = Go/No-Go test _ Reaction time; STROOP_RI = Stroop interference test _Reading-interference; STROOP_NI = Stroop interference test _Naming-interference; BIS = Barratt Impulsivity Scale, BIS_AI = attentional impulsivity, BIS_MI = motor impulsivity, BIS_NI = non-planning impulsivity.

Descriptive statistics and univariate results for the total group (*N* = 654) of individuals are presented in Table 2. Compared to test norms, the number of individuals with “below average” impulsivity performance or self-reports had a large range, from 6.4% to 94.2% per variable for the entire sample. The highest impulsivity rates were found in the self-reports, with motor and attention impulsivity showing below average scores of up to 88.2% and 94.2%, respectively, while non-planning impulsivity showed a smaller proportion of 25.7%. In the performance tests, the highest proportions of below-average impulsivity scores were found in the commission errors of the vigilance test (31.8%). Reaction time variables of impulsivity measures revealed lower proportions of “below average” performance (6.4%–17.0%). Visual inspection of group differences in the proportions of “below average” suggested marginally higher impulsivity proportions in the ADHD group for over half of the measures (7/9), particularly in WAFV omissions and INHIB commissions, though formal statistical comparisons were not conducted (Figure 1).

### Network Analysis

**Figure 2.**
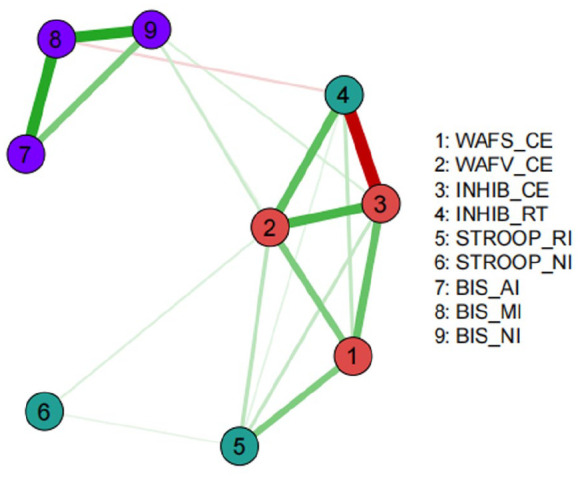
Network of Impulsivity Measures of Adults with ADHD on Subsample 1 (*n* = 279). *Note.* Nodes represent neuropsychological assessment variables. Neuropsychological assessment variables derived from the same assessment are color-coded uniformly. The edges connecting the nodes depict regularized partial Spearman correlations. Thicker and more intensely colored edges signify stronger absolute correlations. Green edges denote positive correlations, whereas red edges represent negative correlations. WAFS_CE = Perceptual and Attention Functions Test (Selective Attention)_Commission errors; WAFV_CE = Perceptual and Attention Functions Test (Vigilance)_Commission errors; INHIB_CE = Go/No-Go test_Commission errors; INHIB_RT = Go/No-Go test_ Reaction time; STROOP_RI = Stroop interference test_Reading-interference; STROOP_NI = Stroop interference test_Naming-interference; BIS = Barratt Impulsivity Scale, BIS_AI = attentional impulsivity, BIS_MI = motor impulsivity, BIS_NI = non-planning impulsivity.

The item-network estimation of the nine impulsivity items was conducted in subsample 1 (*n* = 279), as illustrated in Figure 2. The impulsivity item network was composed of 19 edges (17 positive and 2 negative), revealing multiple connections between the different items (nodes) and especially between items within the same impulsivity domain (i.e., 7–8; 2–3). A visual inspection of the network figure reveals that items 7, 8, and 9 (self-reports factor) form a separate group from items 1, 2, and 3 (commission errors factor) and items 4, 5, and 6 (reaction times factor). However, the interconnections within the reaction times factor were notably weaker compared to those within the self-reports and commission errors factors. Furthermore, based on the results presented in Figure 2, we learn that items 7, 8, and 9 exhibit a relatively stronger level of cohesion compared to items 1, 2, and 3, and items 4, 5, and 6. Interestingly, item 4 (RT of the Go/No-Go test) appears to be more closely integrated in the group of items 1, 2, and 3, collectively representing the commission errors factor. This observation leads to the post hoc proposed factor structure of allocating the reaction time of the Go/No-Go test to the factor with commission errors. In addition, the overall network configuration supports a two-group network, distinguishing performance-based measures (commission errors factor and reaction times factor) from self-report-based measures.

**Figure 3.**
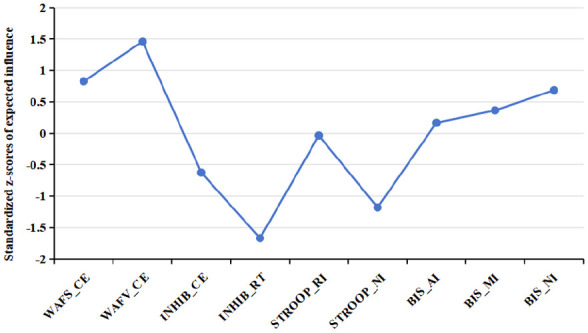
Node Expected Influence on Subsample 1 (*n* = 279). *Note*: Higher standardized z-scores indicate higher expected influence, and nodes with higher expected impact have closer and stronger relationships with other neuropsychological test variables in the network. WAFS_CE = Perceptual and Attention Functions Test (Selective Attention)_Commission errors; WAFV_CE = Perceptual and Attention Functions Test (Vigilance)_Commission errors; INHIB_CE = Go/No-Go test_Commission errors; INHIB_RT = Go/No-Go test_Reaction time; STROOP_RI = Stroop interference test_Reading-interference; STROOP_NI = Stroop interference test_Naming-interference; BIS = Barratt Impulsivity Scale, BIS_AI = attentional impulsivity, BIS_MI = motor impulsivity, BIS_NI = non-planning impulsivity.

**Figure 4.**
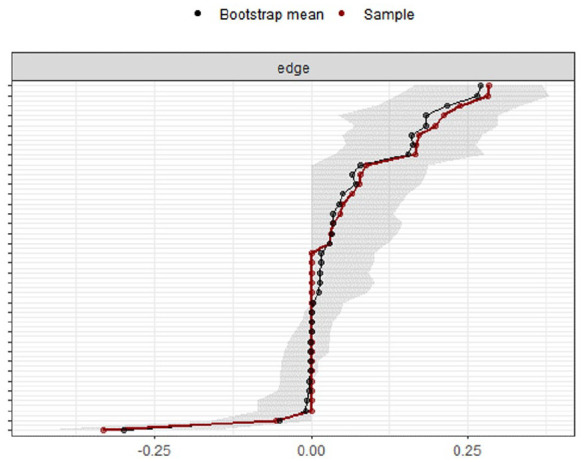
Edge Weight Accuracy Estimation on Subsample 1 (*n* = 279). *Note*: Bootstrapped CIs of estimated edge weights for the estimated network of two samples are shown. Each horizontal line represents one edge of the network, ordered from the edge with the highest edge weight to the edge with the lowest edge weight. The red line indicates the sample values of edge weights, and the black line indicates the Bootstrap mean values of edge weights. The gray area indicates the bootstrapped CIs. The y-axis labels have been removed to avoid cluttering.

Node centrality estimations are presented in Figure 3. Nodes with high expected influence are mainly the commission errors of selective attention and vigilance tests. The edge weight accuracy estimation revealed moderate confidence intervals, indicating that the rank order of edge weights was accurately estimated (Figure 4). Furthermore, the node centrality estimation revealed CS coefficients of 0.52 for subsample 1, indicating the order of node centrality measures was very stable. Thus, reliable and stable network analysis visually confirmed current results.

### Confirmatory Factor Analysis

Measures of fit for the one-, two-, and three-factor CFA models on subsample 2 (*n* = 375) are presented in Table 3. The approximate model fit indices (χ^2^/*df*, CFI, RMSEA, and TLI) of two- and three-factor models were adequate but poor for the one-factor model. The three-factor solution fits best, surpassing both the one- and two-factor models across all fit indices. Moreover, a statistically significant reduction in chi-square was observed when we moved from the one-factor model to the two-factor model, △χ^2^ = 56.54, *p* = 0.019, as well as when we moved from the two-factor model to the theoretical three-factor model, △χ^2^ = 18.26, *p* = 0.049. Thus, results from the CFA indicate that the three-factor solution best accounted for the structure and organization of impulsivity measures grouped by self-reports, commission errors, and reaction times. Based on the results of the network analysis, an alternative three-factor model was proposed, which was tested for its factor structure in CFA. Four fit indices were indicative of good/acceptable model fit according to the recommended cutoff (see Table 3). However, the alternative three-factor model did not improve the fit compared to the initially proposed three-factor model. More specifically, the results provided positive evidence for the superiority of the conceptually derived three-factor model, as indicated by a lower BIC (BIC = 9400.98) compared to the alternative three-factor model (BIC = 9404.35), with a △BIC of 3.4.

### Influence of Potential Invalid Data on Network and Factor Structure

Repeating the network analysis on the subsets of individuals after removing cases with probable underperformed test results (i.e., subset 1, *N* = 544), probable inconsistently reported symptoms (subset 2, *N* = 501), and probable overreported symptoms (subset 3, *N* = 359) revealed consistent results across all exclusion criteria. The network structure, allocation, cohesion, and centrality of each item remained largely unaffected by the exclusion of probable invalid data (see Figures S1), with results consistently supporting the conceptual distinction between two-core network clusters: a performance-based cluster and a self-report cluster. Nodes with high expected influence are mainly the commission errors of the selective attention and vigilance test (Figure S2), indicating their central role in the impulsivity network. The data in Figure S3 show that the rank order of edge weights was accurately estimated. Furthermore, the edge weight accuracy estimation on the three subsets revealed moderate confidence intervals. Node centrality estimation showed a stable rank order across these subsets, with CS coefficients of 0.67 for subset 1, 0.67 for subset 2, and 0.44 for subset 3, respectively.

Similarly, CFA conducted on subsets of participants without indicators of probable invalid performance or symptom reporting consistently showed that the three-factor model provided better overall fit than the one- and two-factor alternatives. In both subset 1and subset 2 (*N* = 544 and *N* = 501), chi-square difference tests confirmed statistically significant improvements in fit for the three-factor model over nested alternatives. In the most conservative subset 3, based on probable symptom overreporting (*N* = 359), the three-factor model again showed the best fit indices, although the advantage over the two-factor model did not reach statistical significance (Δχ² = 5.66, *p* = .059). The detailed fit indices and model comparisons are presented in Table S1.

### Multiple Linear Regression

Results of the multiple regression analysis are presented in Table 4 and reveal that models predicting commission errors (*F* = 4.15, *p* < .001) and symptom reports (*F* = 55.38, *p* < .001) were statistically significant. The strongest predictive value was found for the model predicting symptom reports of impulsivity with 46% explained variance. Hyperactive-impulsive symptoms (β = .0427, *SE* = .0035, *t* = 12.195, *p* < .001) and inattention symptoms (β = .0212, *SE* = .0029, *t* = 7.392, *p* < .001) emerged as the strongest predictors, with higher symptom severity associated with increased impulsivity scores. In addition, sensation seeking (β = .0671, *SE* = .0167, *t* = 4.022, *p* < .001) and sex (β = .1009, *SE* = .0380, *t* = 2.652, *p* = .008) were also significant predictors, suggesting that males and individuals with higher sensation-seeking tendencies reported greater impulsivity. The model predicting impulsivity in commission errors explained only 6% of the variance, with higher-hyperactive-impulsive symptoms (β = .0595, *SE* = .0158, *t* = 3.762, *p* < .001) significantly predicting impulsivity in commission errors. Although the overall model predicting impulsivity based on reaction time was not statistically significant (*F* = 1.05, *p* = .401), inattention symptoms emerged as a statistically significant individual predictor (β = .0015, *SE* = .0007, *t* = 1.98, *p* = .048).

## Discussion

This study conducted advanced statistical analysis on a standard neuropsychological battery from a large cohort of individuals at an ADHD outpatient assessment clinic. Our goal was to clarify the factorial structure of impulsivity measures in these routine assessments, providing clearer guidance for clinicians and researchers in interpreting impulsivity measures.

In the univariate analysis of impulsivity scores based on test norms, we observed the highest levels of clinically significant scores (indicated by “below average” scores) in self-reports of motor and attentional impulsivity, with notably lower proportions in non-planning impulsivity. While a non-trivial proportion of individuals also displayed clinically relevant impulsivity scores in commission error variables, reaction time variables did not reveal significantly slower responses in a smaller proportion of individuals. Elevated levels of impulsivity in an adult ADHD referral context were expected and are consistent with a large body of research (Crunelle et al., 2013; Huang-Pollock et al., 2012; Lopez et al., 2015; Rasouli Mahin et al., 2020). Notably, and in line with previous studies (Guo, Fuermaier, Koerts, Mueller, Diers, et al., 2021; Guo, Fuermaier, Koerts, Mueller, Mette, et al., 2021), none of these measures appear to be useful for diagnostic purposes as the ADHD group only differed marginally from individuals without an ADHD diagnosis.

Independent CFA confirmed our assumptions of a three-factor structure of impulsivity, representing (a) self-reported information on impulsivity, as well as (b) commission errors and (c) reaction time variables in neuropsychological tests. This factor structure derived from this study specifically applies to routine neuropsychological batteries as they are typically used in this context. The factor on self-reported impulsivity included all subscales of the BIS, which is a motor-, attentional, and non-planning component of impulsivity, which collectively represent trait-dependent impulsivity. Importantly, these subscales are designed to measure interrelated facets of impulsivity, and thus, moderate intercorrelations among them are theoretically expected (Patton et al., 1995; Stanford et al., 2009). The overlap suggests that while the subscales capture distinct behavioral manifestations (e.g., impulsive actions vs. decision-making), they likely share common underlying psychological mechanisms (Cyders & Coskunpinar, 2011; Reise et al., 2013). Previous psychometric studies of the BIS have suggested various factor structures, including a two-factor model (Reise et al., 2013), a three-factor model (Fossati et al., 2001), or even questioned the existence of a stable factor structure for this scale (Khemiri et al., 2021; Vasconcelos et al., 2012). For clinical practice, our findings suggest that the total score of the BIS can be used as a reliable and practical measure of self-reported impulsivity, particularly in the assessment of adult ADHD. This approach simplifies clinical interpretation by providing a unified measure of impulsivity traits, reducing the need to differentiate between multiple dimensions based on self-reported information. While the results supported a coherent self-report impulsivity factor encompassing all BIS subscales, it was important to consider that these subscales come from the same instrument, which may introduce shared method variance. The shared variance can inflate intercorrelations and factor cohesion, potentially conflating measurement artifacts with true construct-level unity. Therefore, our findings regarding the BIS subscale cohesion should be interpreted with caution.

The two other factors of impulsivity (i.e., commission errors and reaction times) jointly capture aspects of state-dependent impulsivity and can be assessed by neuropsychological performance tests. These tests have in common that they measure the inability to withhold a prepotent response and require a trade-off between accuracy and speed. Data analysis confirmed our assumption that commission errors form a separate group (as shown in network analysis) and consistently load onto a single factor in CFA. In contrast, variables of the reaction-time factor of impulsivity showed weaker interconnections compared to the self-reported factor and commission errors factor, indicating that reaction time measures may be less stable or more context-dependent, which could explain their lower centrality in the network. The observed instability in the reaction-time factor may also be partially attributable to the limited sample size as a consequence of independent network analysis and CFA. In supplementary analysis, we showed that a larger sample yielded a more robust and stable network per factor, as evidenced by the stronger and more consistent interconnections observed in the full sample compared to the subsample analyses (see Figure S4, S5, and S6). This aligns with the observation in the network analysis of subsample 1 that one of the reaction time variables (RT of Go/No-Go) may be better allocated to the commission errors factor, suggesting that reaction times in this specific task may share more variance with commission errors than with other reaction time measures on impulsivity. However, this assumption was not supported in post hoc CFA on a different sample. Furthermore, as another explanation, the weaker interconnections of reaction-time impulsivity measures may express a limited range of scores and performance in the normal range for most participants. This is supported by the fact that we did not observe significantly deviant scores in the clinical group compared to the test norms for these variables. The overall network structure further reinforces a two-group configuration, wherein performance-based measures (commission errors and reaction times) are topologically separated from self-report measures (BIS subscales). Specifically, in the network of subsample 1 (Figure 2), BIS indicators (items 7–9) formed a clearly defined cluster, characterized by high internal cohesion and strong mutual edges, and distinctly separated from the performance-based nodes. This pattern is consistent with trait–state impulsivity frameworks, which differentiate stable self-perceived impulsivity from momentary behavioral expressions (Cyders & Coskunpinar, 2011; Sharma et al., 2014), and reflects the well-documented dissociation in ADHD between subjective symptom reporting and objective task performance (Barkley & Fischer, 2011; Barkley & Murphy, 2010; Biederman et al., 2008; Butzbach et al., 2019; Fuermaier et al., 2015; Guo, Fuermaier, Koerts, Mueller, Mette, et al., 2021; Kallweit et al., 2020; Nelson & Lovett, 2019).

The factor structure of impulsivity presented and confirmed in this study demonstrates the cross-task consistency of impulsivity measures. Based on this, we conclude that assessing impulsivity in adults with ADHD may not require repeated measurement of the same variables across multiple tasks, nor repeated univariate interpretation of the same test variables. A more streamlined assessment and interpretation could save time and resources, enhancing patient compliance and the practicality of clinical testing. This may also be relevant as the clinical assessment could rely on those measures with the best psychometric properties and omit those with unsatisfactory properties (e.g., low test–retest reliability; see Kindlon et al., 1995).

Numerous studies have identified risk factors for impulsivity, including male sex, younger adults, more severe symptoms of ADHD, the presence of comorbid psychiatric disorders (such as addiction or mood disorders), or high levels of sensation-seeking tendency (Daurio et al., 2018; Hasson & Fine, 2012; Skirrow et al., 2009; Steinberg et al., 2008; Zhuang et al., 2025). Notably, while addiction disorders have frequently been linked to impulsivity in prior research, no significant association was observed in our sample. This may be due to the clinical characteristics and referral patterns of our sample, which likely reduced variance in impulsivity and led to underreporting or underdiagnosis of certain comorbidities. However, differential relationships between demographic and clinical information on the one side and distinct components of impulsivity on the other side remained unclear (Cross et al., 2011; Stanford et al., 2009). Our regression models revealed that demographic and clinical information was most powerful in predicting self-reported impulsivity, with 46% explained variance. Inattentive and hyperactive-impulsive symptom severity emerged as the strongest predictors. This finding can be explained by the fact that self-reports of impulsivity are closely tied to the core symptomatology of ADHD, particularly inattention and hyperactivity–impulsivity (Barkley & Murphy, 2006; Miller et al., 2010). In addition, sensation-seeking tendencies and, with a smaller effect size, male sex significantly predicted self-reported impulsivity, findings that are consistent with previous research (Harden & Tucker-Drob, 2011; Lage et al., 2013). These findings underscore the importance of considering both clinical and demographic characteristics in the clinical evaluation of adult ADHD and associated pathology. In contrast to the relatively large proportion of explained variance of self-reported impulsivity, a significant yet small proportion of variance (6%) could be explained for commission error impulsivity (with hyperactive-impulsive symptoms being the sole significant predictor), and no significant model was obtained for reaction time variables (less than 2% explained variance). The smaller proportion of explained variance of performance-based impulsivity measures is not surprising, given that the nature of the assessment of (self-reported) ADHD symptom severity and sensation seeking aligns better with self-reported impulsivity than with performance measures (Cyders & Coskunpinar, 2012; Fuermaier et al., 2015; Kräplin et al., 2014; Reynolds et al., 2008). The differential predictive models for the three impulsivity factor scores further emphasize the need to interpret these domains independently in clinical assessments. These findings not only highlight the differential predictive utility of clinical and demographic variables across impulsivity domains but also align with the observed two-group network in the network analysis. Specifically, the regression models revealed robust shared variance between clinical predictors (e.g., ADHD symptom severity and sensation seeking) and the self-report measures, while showing no or only minimal shared variance with performance-based measures. This pattern of associations lends further support to a conceptual distinction between self-reported and performance-based impulsivity, consistent with the two-group network configuration observed in our structural analyses.

### Limitations and Future Directions

Several limitations must be acknowledged in the interpretation of the present findings. First, impulsivity measures used in this study were derived from a routine neuropsychological battery as it is typically applied in this context; however, the battery has not been composed for this study purpose. For instance, additional behavioral tasks (e.g., delay discounting, risk-taking paradigms), physiological measures (e.g., heart rate variability), or imaging techniques could provide further insights into the factor structure and interrelatedness of impulsivity in this assessment context. The current findings are restricted to routine assessments of this kind, as impulsivity is an inherently heterogeneous construct, and the number of factors depends on the number and types of measures. Future research should aim to include a broader range of self-report impulsivity measures from different instruments to clarify whether the observed factor structure generalizes beyond the BIS and to more precisely separate construct variance from method variance. This would strengthen the evidence for a unified self-reported impulsivity factor and enhance understanding of the multifaceted nature of impulsivity in clinical populations. Second, prediction models of demographic and clinical information on impulsive behavior would be particularly interesting in longitudinal study designs to determine risk or protective factors of impulsive behavior later in time and to explain how impulsive behavior evolves and shapes in response to ADHD persistence or remission. Prediction models could include additional information that may be relevant for this context, for example, retrospective childhood behavior, parent (or significant other) reports, or cognitive impairment. Third, clinical application of the derived three-factor structure can be facilitated by demonstrating the suitability of the impulsivity measures as treatment outcomes and by determining distinct subgroups of individuals (e.g., with cluster analysis) who show particular symptom constellations and marked scores of impulsivity.

## Conclusions

Adults evaluated for ADHD typically exhibit elevated scores across various indicators of impulsive behavior. The present study examines impulsivity measures derived from a comprehensive routine neuropsychological battery in an ADHD outpatient assessment context, revealing a robust factor structure comprising (a) self-reports, (b) commission errors, and (c) reaction times in neuropsychological performance tests. This structure of impulsivity measures can aid clinicians and researchers in selecting and interpreting the most relevant measures. We confirm that self-reports should be interpreted independently of performance tests and demonstrate cross-task consistency between commission errors and reaction times. While symptom severity and other clinical and demographic variables are closely linked to self-reported impulsivity, performance-based indicators of impulsivity are less predictably influenced by demographic or clinical features.

## Supplemental Material

sj-docx-1-asm-10.1177_10731911251365744 – Supplemental material for Indicators of Impulsivity in Routine Clinical Assessment of Adult ADHD Supplemental material, sj-docx-1-asm-10.1177_10731911251365744 for Indicators of Impulsivity in Routine Clinical Assessment of Adult ADHD by Hui Dong, Anselm B.M. Fuermaier, Janneke Koerts, Gerdina H.M. Pijnenborg, Nana Guo, Ragnar Schwierczok, Norbert Scherbaum and Bernhard W. Müller in Assessment
