# Supplementary material for: Indicators of Impulsivity in Routine Clinical Assessment of Adult ADHD
Source: Assessment. 2025 Sep 16;33(6):982–99. doi: 10.1177/10731911251365744 (PMC13379612; doi:10.1177/10731911251365744)
Supplement: sj-docx-1-asm-10.1177_10731911251365744 – Supplemental material for Indicators of Impulsivity in Routine Clinical Assessment of Adult ADHD [file sj-docx-1-asm-10.1177_10731911251365744.docx]

**Supplementary file**

**Table S1. CFA model fit statistics (one-, two-, and three-factor solutions) on the subsets of data after exclusion of probable underperformed test results (WAFV > 7), probable inconsistently reported symptoms (CAARS inconsistency > 8), and probable overreported symptoms (CAARS DSM T > 80)**

| **WAFV_omissions ≥ 7, N = 544** | | | | | | |
| --- | --- | --- | --- | --- | --- | --- |
| Model | χ^2^ | χ^2^/*df* | RMSEA | CFI | TLI | Model comparisons |
| Three-factor Model | 39.312 | 1.64 | 0.033 | 0.959 | 0.938 | Three-factor vs. Two-factor  △χ2 = 14.33, *p* < .001 |
| Three-factor model (post-hoc) | 42.566 | 1.77 | 0.037 | 0.949 | 0.923 |  |
| Two-factor Model | 65.852 | 2.53 | 0.058 | 0.863 | 0.810 | One-factor vs. Two-factor  △χ2 = 19.06, *p* < .001 |
| One-factor Model | 141.678 | 5.25 | 0.083 | 0.710 | 0.613 |  |
| **CAARS_inconsistency scores ≥ 8, N = 501** | | | | | | |
| Goodness of fit | χ^2^ | χ^2^/*df* | RMSEA | CFI | TLI | Model comparisons |
| Three-factor Model | 40.359 | 1.68 | 0.036 | 0.964 | 0.946 | Three-factor vs. Two-factor  △χ2 = 12.01, *p* = .002 |
| Three-factor model (post-hoc) | 45.641 | 1.90 | 0.040 | 0.955 | 0.932 |  |
| Two-factor Model | 57.279 | 2.20 | 0.046 | 0.936 | 0.912 | One-factor vs. Two-factor  △χ2 = 34.96, *p* < .001 |
| One-factor Model | 169.969 | 6.30 | 0.097 | 0.701 | 0.602 |  |
| **CAARS_DSM total T scores ≥ 80, N = 359** | | | | | | |
| Goodness of fit | χ^2^ | χ^2^/*df* | RMSEA | CFI | TLI | Model comparisons |
| Three-factor Model | 31.987 | 1.33 | 0.036 | 0.921 | 0.881 | Three-factor vs. Two-factor  △χ2 = 5.66, *p* = .059 |
| Three-factor model (post-hoc) | 34.421 | 1.43 | 0.039 | 0.911 | 0.866 |  |
| Two-factor Model | 44.383 | 1.71 | 0.049 | 0.847 | 0.788 | One-factor vs. Two-factor  △χ2 = 13.56, *p* < .001 |
| One-factor Model | 95.584 | 3.54 | 0.079 | 0.583 | 0.443 |  |

*Note:* WAFV = Perceptual and Attention Functions Test_Vigilance; CAARS = Conners’ Adult ADHD Rating Scales; CFA = confirmatory factor analysis; RMSEA = root-mean-square error of approximation; CFI = comparative fit index; TLI = Tucker–Lewis index; Model-data fit is considered acceptable when χ^2^/*df* ranges from 2 to 5, RMSEA is below 0.06, and CFI and TLI estimates exceed 0.80; A fit is considered good when χ^2^/*df* close to 1, and CFI, TLI estimates surpass 0.95 (Hox et al., 2017).

**C**

**B**

**A**


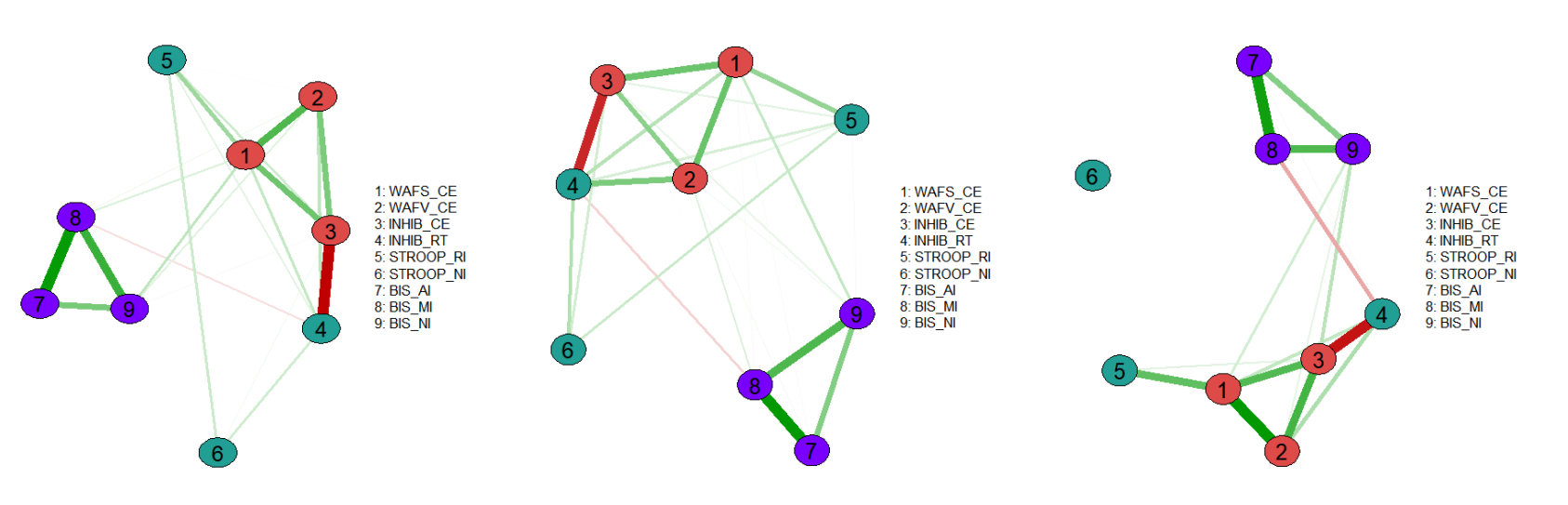


**Figure S1. Network of impulsivity measures after exclusion of probable underperformed test results (WAFV > 7), probable inconsistently reported symptoms (CAARS inconsistency > 8), and probable overreported symptoms (CAARS DSM T > 80)**

*Note*: Nodes represent neuropsychological test variables. Neuropsychological test variables derived from the same assessment are color-coded uniformly. The edges connecting the nodes depict regularized partial Spearman correlations. Thicker and more intensely colored edges signify stronger absolute correlations. Green edges denote positive correlations, whereas red edges represent negative correlations. WAFS_CE = Perceptual and Attention Functions Test (Selective Attention)_Commission errors; WAFV_CE = Perceptual and Attention Functions Test (Vigilance)_Commission errors; INHIB_CE = Go/No-Go test_Commission errors; INHIB_RT = Go/No-Go test_Reaction time; STROOP_RI = Stroop interference test_Reading-interference; STROOP_NI = Stroop interference test_Naming-interference; BIS = Barratt Impulsivity Scale, BIS_AI = attentional impulsivity, BIS_MI = motor impulsivity, BIS_NI = non-planning impulsivity; CAARS = Conners’ Adult ADHD Rating Scales. Visualization of impulsivity network structures after applying different data-cleaning criteria: (A) excluding participants with probable underperformed test results (WAFV_Omissions ≥ 7; N = 544), (B) excluding those with probable inconsistently reported symptoms (CAARS Inconsistency Index ≥ 8; N = 501), and (C) excluding those with probable overreproted symptoms (CAARS DSM Total T-score ≥ 80; N = 359).

**C**

**B**

**A**


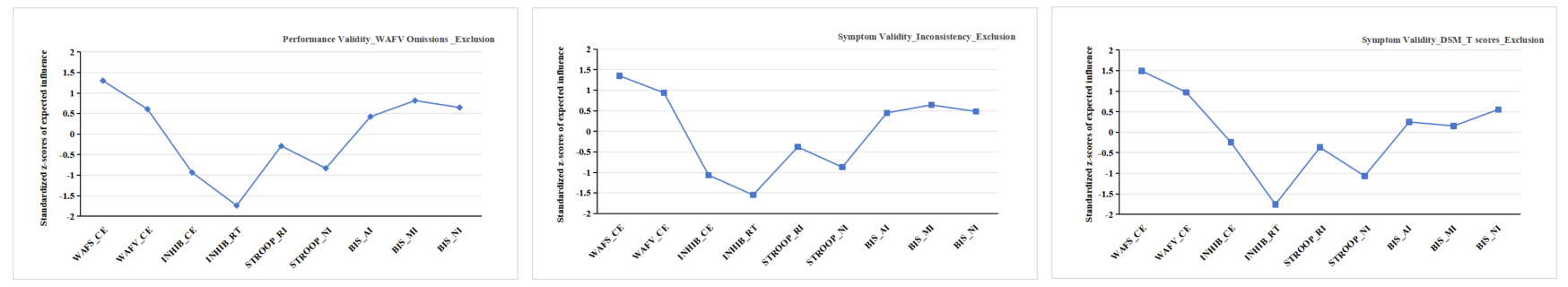


**Figure S2. Node expected influence in network models based on data subsets after exclusion of probable underperformed test results (WAFV > 7), probable inconsistently reported symptoms (CAARS inconsistency > 8), and probable overreported symptoms (CAARS DSM T > 80)**

*Note*: Higher standardized z-scores indicate higher expected influence, and nodes with higher expected impact have closer and stronger relationships with other neuropsychological test variables in the network. WAFS_CE = Perceptual and Attention Functions Test (Selective Attention)_Commission errors; WAFV_CE = Perceptual and Attention Functions Test (Vigilance)_Commission errors; INHIB_CE = Go/No-Go test _Commission errors; INHIB_RT = Go/No-Go test_ Reaction time; STROOP_RI = Stroop interference test_Reading-interference; STROOP_NI = Stroop interference test_Naming-interference; BIS = Barratt Impulsivity Scale, BIS_AI = attentional impulsivity, BIS_MI = motor impulsivity, BIS_NI = non-planning impulsivity. CAARS = Conners’ Adult ADHD Rating Scales. (A) excluding participants with probable underperformed test results (WAFV_Omissions ≥ 7; N = 544), (B) excluding those with probable inconsistently reported symptoms (CAARS Inconsistency Index ≥ 8; N = 501), and (C) excluding those with probable overreproted symptoms (CAARS DSM Total T-score ≥ 80; N = 359).

**C**

**A**

**B**


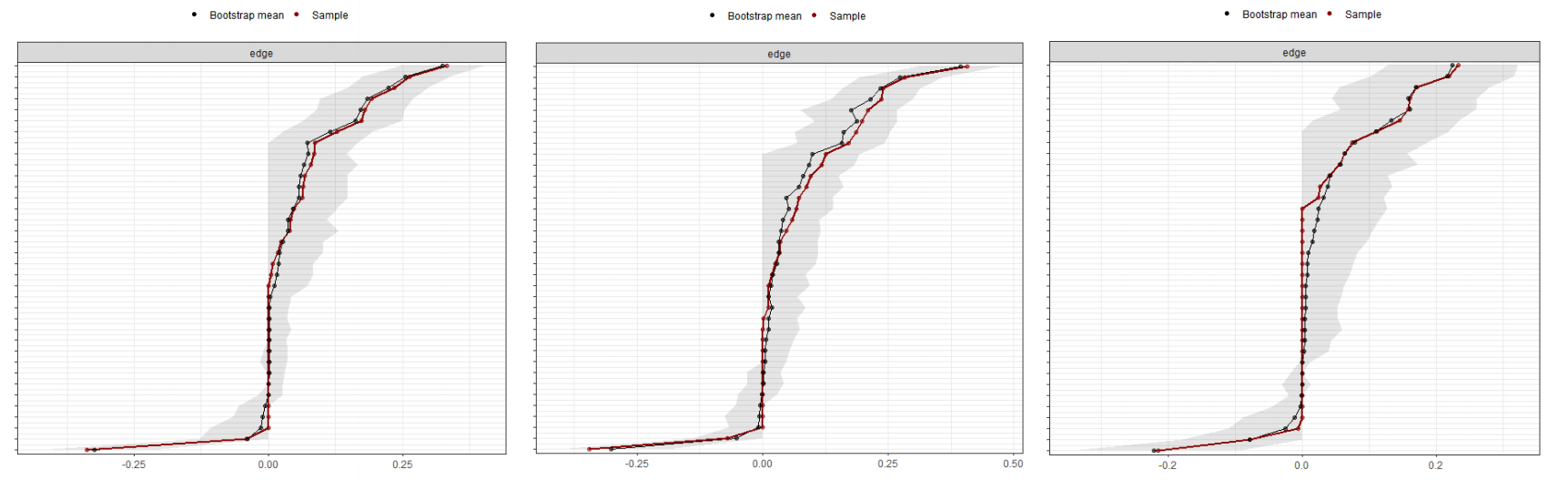


**Figure S3. Edge weight accuracy estimation in network models based on data subsets after exclusion of probable underperformed test results (WAFV > 7), probable inconsistently reported symptoms (CAARS inconsistency > 8), and probable overreported symptoms (CAARS DSM T > 80)**

*Note*: Bootstrapped CIs of estimated edge-weights for the estimated network of the valid data. Each horizontal line represents one edge of the network, ordered from the edge with the highest edge-weight to the edge with the lowest edge-weight. The red line indicates the sample values of edge weights and the black line indicates the Bootstrap mean values of edge weights. The gray area indicates the bootstrapped CIs. The y-axis labels have been removed to avoid cluttering. WAFV = Perceptual and Attention Functions Test (Vigilance); CAARS = Conners’ Adult ADHD Rating Scales; (A) excluding participants with probable underperformed test results (WAFV_Omissions ≥ 7; N = 544), (B) excluding those with probable inconsistently reported symptoms (CAARS Inconsistency Index ≥ 8; N = 501), and (C) excluding those with probable overreproted symptoms (CAARS DSM Total T-score ≥ 80; N = 359).


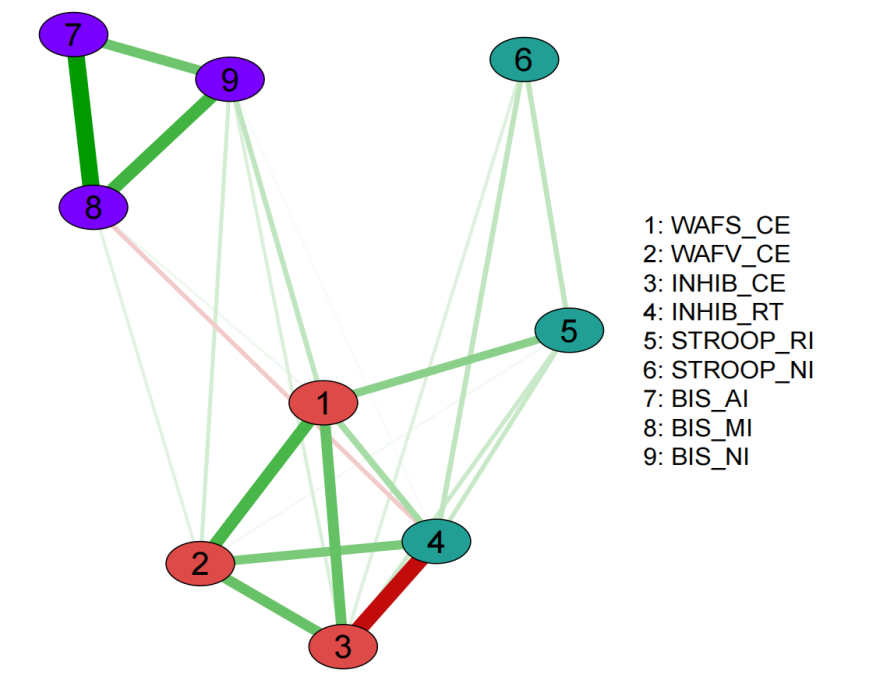


**Figure S4. Network of impulsivity for adults with ADHD on** **the entire sample** **(N = 654)**

*Note.* Nodes represent neuropsychological assessment variables. Neuropsychological assessment variables derived from the same assessment are color-coded uniformly. The edges connecting the nodes depict regularized partial Spearman correlations. Thicker and more intensely colored edges signify stronger absolute correlations. Green edges denote positive correlations, whereas red edges represent negative correlations. WAFS_CE = Perceptual and Attention Functions Test (Selective Attention)_Commission errors; WAFV_CE = Perceptual and Attention Functions Test (Vigilance)_Commission errors; INHIB_CE = Go/No-Go test_Commission errors; INHIB_RT = Go/No-Go test_ Reaction time; STROOP_RI = Stroop interference test_Reading-interference; STROOP_NI = Stroop interference test _Naming-interference; BIS = Barratt Impulsivity Scale, BIS_AI = attentional impulsivity, BIS_MI = motor impulsivity, BIS_NI = non-planning impulsivity.


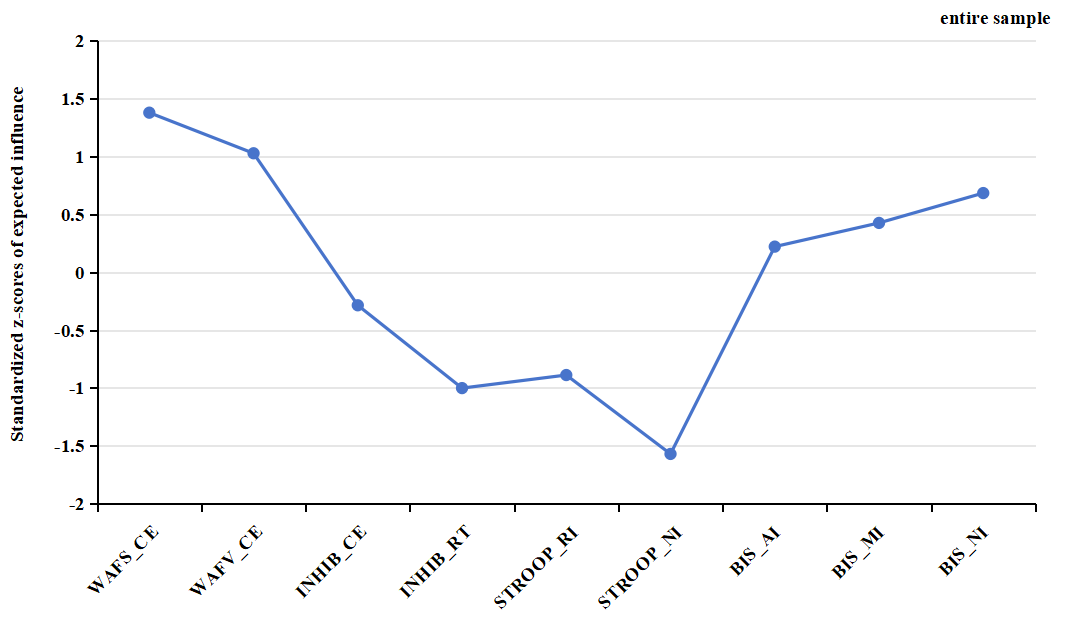


**Figure S5. Node Expected Influence on the entire sample (N = 654)**

*Note:* Higher standardized z-scores indicate higher expected influence and nodes with higher expected impact have closer and stronger relationships with other neuropsychological test variables in the network. WAFS_CE = Perceptual and Attention Functions Test (Selective Attention)_Commission errors; WAFV_CE = Perceptual and Attention Functions Test (Vigilance)_Commission errors; INHIB_CE = Go/No-Go test_Commission errors; INHIB_RT = Go/No-Go test_Reaction time; STROOP_RI = Stroop interference test_Reading-interference; STROOP_NI = Stroop interference test_Naming-interference; BIS = Barratt Impulsivity Scale, BIS_AI = attentional impulsivity, BIS_MI = motor impulsivity, BIS_NI = non-planning impulsivity.


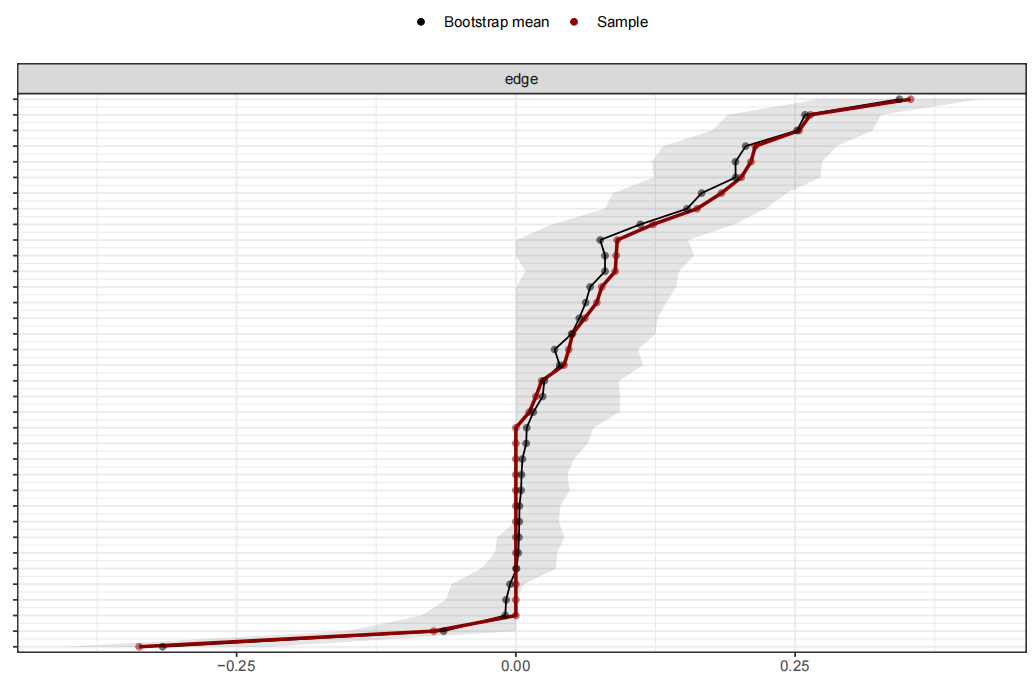


**Figure S6. Edge weight accuracy estimation on the entire sample (N = 654)**

*Note:* Bootstrapped CIs of estimated edge-weights for the estimated network of the entire sample. Note. Each horizontal line represents one edge of the network, ordered from the edge with the highest edge-weight to the edge with the lowest edge-weight. The red line indicates the sample values of edge weights and the black line indicates the Bootstrap mean values of edge weights. The gray area indicates the bootstrapped CIs. The y-axis labels have been removed to avoid cluttering.

**t Scherbaum ^3, 4^,** **Bernhard W. Müller ^3, 4, 5^**

^1^ Department of Clinical and Developmental Neuropsychology, Faculty of Behavioral and Social Sciences, University of Groningen, 9712 TS Groningen, The Netherlands

^2^ Department of Psychology, Faculty of Education, Henan Normal University, Xinxiang, China

^3^ LVR- University Hospital Essen, Department of Psychiatry and Psychotherapy, Faculty of Medicine, University of Duisburg-Essen, Essen, Germany

^4^ Center for Translational Neuro and Behavioral Sciences (C-TNBS), Faculty of Medicine, University of Duisburg-Essen, Essen, Germany

^5^ Department of Psychology, University of Wuppertal, Wuppertal, Germany

*Correspondence: Anselm B.M. Fuermaier; Department of Clinical and Developmental Neuropsychology, Faculty of Behavioral and Social Sciences, University of Groningen, Grote Kruisstraat 2/1, 9712 TS Groningen, The Netherlands, Tel.: +31 503639729, [a.b.m.fuermaier@rug.nl](mailto:a.b.m.fuermaier@rug.nl)

## Acknowledgments: We thank all research assistants involved in this project for their support in data collection and processing.

**Funding:** This research was supported by a China Scholarship Council (CSC) scholarship to Hui Dong., grant number 202206990011

**Consent to Participants:** Informed consent was obtained from all individual participants included in the study.

**Conflicts of Interest:** ABMF and JK have contracts with Schuhfried GmbH for the development and evaluation of neuropsychological assessment instruments. ABMF is co-author of the test set “Cognitive Functions ADHD (CFADHD)” that is administered and examined in the present study. The CFADHD is a neuropsychological test battery on the Vienna Test System (VTS), owned and distributed by the test publisher Schuhfried GmbH.
